# Predictors of falls and mortality among elderly adults with traumatic brain injury: A nationwide, population-based study

**DOI:** 10.1371/journal.pone.0175868

**Published:** 2017-04-21

**Authors:** Wayne W. Fu, Terence S. Fu, Rowan Jing, Steven R. McFaull, Michael D. Cusimano

**Affiliations:** 1 Albany Medical College, Albany, NY, United States of America; 2 Division of Neurosurgery, Department of Surgery, St. Michael’s Hospital; Injury Prevention Research Office, Li Ka Shing Knowledge Institute, Keenan Research Centre; University of Toronto; Toronto, Canada; 3 Injury Section, Health Surveillance and Epidemiology Division, Public Health Agency of Canada, Ottawa, Ontario, Canada; 4 Dalla Lana School of Public Health, University of Toronto, Toronto, Canada; University of Pennsylvania, UNITED STATES

## Abstract

**Background:**

Elderly adults are at particular risk of sustaining a traumatic brain injury (TBI), and tend to suffer worse outcomes compared to other age groups. Falls are the leading cause of TBI among the elderly.

**Methods:**

We examined nationwide trends in TBI hospitalizations among elderly adults (ages 65 and older) between April 2006 and March 2011 using a population-based database that is mandatory for all hospitals in Canada. Trends in admission rates were analyzed using linear regression. Predictors of falls and in-hospital mortality were identified using logistic regression.

**Results:**

Between 2006 and 2011, there were 43,823 TBI hospitalizations resulting in 6,939 deaths among elderly adults in Canada. Over the five-year study period, the overall rate of TBI admissions increased by an average of 6% per year from 173.2 to 214.7 per 100,000, while the rate of fall-related TBI increased by 7% annually from 138.6 to 179.2 per 100,000. There were significant trends towards increasing age and comorbidity level (p<0.001 and p = 0.002). Advanced age, comorbidity, and injury severity were independent predictors of both TBI-related falls and mortality on multivariate analysis.

**Conclusion:**

Prevention efforts should be targeted towards vulnerable demographics including the “older old” (ages 85 and older) and those with multiple medical comorbidities. Additionally, hospitals and long-term care facilities should be prepared to manage the burgeoning population of older patients with more complex comorbidities.

## Introduction

Traumatic brain injury (TBI) is a major cause of mortality and morbidity worldwide. [1–4] In the United States alone, an estimated 1.7 million people sustained a TBI annually, resulting in 275,000 hospitalizations and 52,000 deaths, and costing approximately $76.5 billion from direct and indirect medical fees in 2010. [5–6] TBI has a similar presence in Canada proportionally with an estimated 25,000 hospitalizations for TBI each year, resulting in over 10,000 deaths. [3]

Although TBI afflicts people of all ages, the elderly population is at particular risk. [2–5,7–9] In the past few decades, TBI rates have declined among young adults, potentially due to greater public awareness and improved preventative measures; in contrast, elderly adults continue to experience the highest and fastest growing TBI rates of any age group. [2,3,7,10,11] In addition, elderly populations are known to suffer worse outcomes and require prolonged recovery compared to other age groups even after controlling for injury severity. [12–14]

Among elderly adults, falls are the most common cause of TBI, representing 50% to 80% of injuries in this population. [3,5,7,8,11] Physiologic age-related changes, medical comorbidities, and a propensity towards polypharmacy all contribute to the increased risk of mechanical injury among the elderly. [15,16] By 2031, it is estimated that one in four Canadians and one in five Americans will be seniors. [17,18] Therefore, understanding the factors impacting TBI-related falls and outcomes will become increasingly important in the development of preventative efforts targeted to this vulnerable and growing population.

There are limited population-based studies examining recent trends in TBI among the elderly, and none, to our knowledge, that have identified predictors of falls and mortality among this vulnerable population. The purpose of our study is three-fold: to (1) investigate trends in elderly TBI-related hospitalizations and in-hospital mortality, (2) identify factors impacting falls and mortality in the elderly, and (3) discuss implications for public health policy and prevention.

## Methods

### Study design and population

This was a national, population-based descriptive study of TBI hospitalizations among elderly adults (age 65 and older) over a five-year period between April 1, 2006 and March 31, 2011. Incidence data were obtained from the Hospital Morbidity Database (HMDB), a mandatory reporting database of hospital admission records for 692 acute care institutions across Canada. Each hospital record includes information on age, sex, mechanism of injury, admission source, length of stay, and up to ten diagnosis codes. Several chart re-abstraction studies have verified the high quality of data maintained in these datasets, with the most recent study reporting 86% agreement for the most responsible diagnosis between database records and hospital charts. [19] Approval for this study was obtained from the Research Ethics Board at St. Michael’s Hospital.

TBI was defined using the following International Classification of Diseases, Tenth Revision (ICD-10) codes: open wound of head [S01(.7,.8,.9)], fracture of skull and facial bones [S02(.0,.1,.7-.9)], intracranial injury (S06.0-S06.9), crushing injury of head [S07(.1,.8,.9)], unspecified injury of head (S09.7-S09.9), injuries involving head with neck (T02.0,T04.0,T06.0), and sequelae of injuries of head [T90(.2,.5,.8,.9)]. Although the Centers for Disease Control and Prevention (CDC) includes additional ICD-10 codes in their definition of TBI mortality, we chose a more conservative set of codes to capture both TBI mortality and morbidity. [3,5,20,21] Patients who registered but left without being seen were excluded. Mechanisms of injury were defined using the CDC’s External Cause of Injury Matrix and collapsed into several main categories: falls, struck by/against an object, motor vehicle collisions, and other causes. [22]

The Charlson Comorbidity Index (CCI) is a widely-used indicator of disease burden which identifies 19 clinical conditions that are significant predictors of mortality, including congestive heart failure, liver disease, and renal disease. [23] A CCI was calculated for each hospital admission using a validated ICD-10 coding algorithm. [24] An injury severity score was also assigned to each hospitalization using the International Classification of Diseases Injury Severity Score (ICISS), a validated measure that has been used extensively in trauma research.[25,26] The ICISS measures the survival probability of each patient on a scale of 0 to 1; lower ICISS scores are associated with a higher probability of death, and therefore indicate greater injury severity. We classified cases into four severity categories based on the 25^th^, 50^th^, 75^th^ percentiles of all ICISS scores.

### Statistical analysis

Descriptive statistics were used to describe the patient population. Hospitalization rates were calculated using population data from Statistics Canada, and reported with 95% confidence intervals (CI). Linear regression was used to evaluate trends in hospitalization rates. A Chi-square test was used to compare (1) survivors versus non-survivors and (2) fall-related TBI versus other mechanisms of injury. Logistic regression was then used to identify predictors of falls and in-hospital mortality. Factors significant on univariable analysis were entered into a multivariable logistic regression model. Adjusted and unadjusted odds ratios (OR) were calculated with corresponding 95% CIs. Multicollinearity was assessed with a variance inflation factor over 4. All analyses were performed using SAS 9.4 (SAS Institute, Inc., Cary, NC, USA). A p-value of less than 5% was considered significant.

## Results

**Fig 1:**
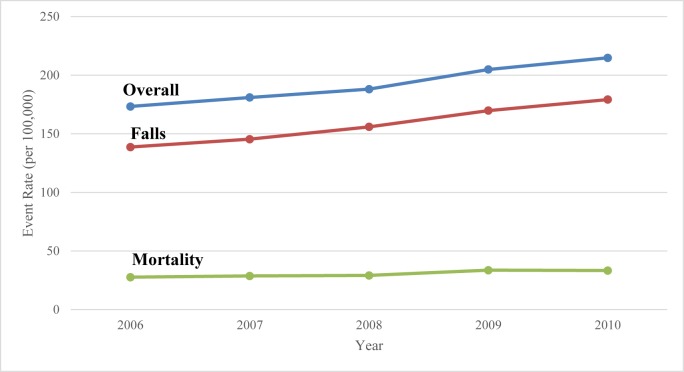
Overall TBI rates of overall hospitalization, mortality, and falls for the elderly population in Canada from 2006/2007-2010/2011.

During the five-year study period, there were 43,823 TBI-related hospitalizations resulting in 6,939 deaths among elderly patients aged 65 and older (Table 1). There was a significant increase in the rate of TBI hospitalizations from 173.2 to 214.7 per 100,000 with an average increase of 6% per year (24% overall increase, p = 0.002; Fig 1 and Table 1). Patients were primarily admitted from the emergency department (86%) or transferred directly from other health care facilities (14%). The majority of patients were discharged home with or without supports services (50%), while the remaining patients were discharged to an inpatient hospital facility (14%), long-term care facility (18%), or other outpatient hospital facilities (2%), or died in hospital (16%). There was a significant increasing trend in the rate of discharge to home, inpatient rehabilitation, and long-term care facilities (p = 0.004, 0.02, and 0.001, respectively).

**Table 1:** Characteristics of hospitalized TBI patients, 2006–2010.

	Incidence		Rate (95% CI) ^*^		Average Percent Change	P-value^†^
	2006	2010	2006	2010		
**Overall**	7467	10299	173.2 (169.3–177.2)	214.7 (210.6–218.9)	6%	0.002
**Male**						
Total	3850	5257	204.8 (198.4–211.3)	247.6 (241.0–254.3)	5%	0.001
65–74	1431	1781	131.9 (125.1–138.7)	144.3 (137.6–151.0)	2%	0.05
75–84	1598	2139	250.1 (237.8–262.3)	309.1 (296.1–322.2)	6%	0.008
85+	821	1337	526.9 (490.9–562.8)	678.4 (642.2–714.7)	7%	0.003
**Female**						
Total	3617	5042	148.8 (144.0–153.7)	188.6 (183.4–193.8)	6%	0.02
65–74	863	1047	72.1 (67.3–76.9)	77.8 (73.1–82.5)	2%	0.26
75–84	1491	1949	169.1 (160.5–177.7)	215.0 (205.5–224.5)	6%	0.01
85+	1263	2046	359.3 (339.5–379.1)	485.9 (464.9–506.9)	8%	0.01
**Mechanism of injury**						
Fall	5975	8595	138.6 (135.1–142.1)	179.2 (175.4–183.0)	7%	0.001
Struck	175	213	4.1 (3.5–4.7)	4.4 (3.8–5.0)	3%	0.49
MVC	834	875	19.4 (18.7–21.4)	18.2 (17.0–19.5)	-1%	0.16
Other^‡^	483	616	11.2 (10.2–12.2)	12.8 (11.8–13.9)	4%	0.33
**CCI**						
1–2	608	695	14.1 (13.0–15.2)	14.5 (13.4–15.6)	1%	0.85
3–4	3646	4570	84.6 (81.9–87.3)	95.3 (92.5–98.0)	3%	0.04
5+	3213	5034	74.5 (72.0–77.1)	105.0 (102.1–107.9)	9%	0.001
**ICISS**						
Below 25th percentile	1840	2538	42.7 (40.7–44.6)	52.9 (50.9–55.0)	6%	0.001
25th to 50th percentile	2034	3241	47.2 (45.1–49.2)	67.6 (65.2–69.9)	9%	< .0001
50th to 75th percentile	1618	2096	37.5 (35.7–39.4)	43.7 (41.8–45.6)	4%	0.005
Above 75th percentile	1975	2424	45.8 (43.8–47.8)	50.5 (48.5–52.6)	3%	0.25
**Length of stay (days)**						
1	1055	1345	24.5 (23.0–26.0)	28.0 (26.5–29.5)	4%	0.05
2–3	1130	1581	26.2 (24.7–27.7)	33.0 (31.3–34.6)	6%	0.024
4–6	1223	1662	28.4 (26.8–30.0)	34.7 (33.0–36.3)	5%	0.007
7–14	1674	2448	38.8 (37.0–40.7)	51.0 (49.0–53.1)	7%	0.005
15–30	1253	1672	29.1 (27.5–30.7)	34.9 (33.2–36.5)	5%	0.01
30+	1132	1591	26.3 (24.7–27.8)	33.2 (31.5–34.8)	6%	0.003
**Admission source**						
Clinic	21	21	0.5 (0.3–0.7)	0.4 (0.3–0.6)	0%	0.25
Direct	981	1455	22.8 (21.3–24.2)	30.3 (28.8–31.9)	8%	0.006
Emergency Department	6457	8818	149.8 (146.2–153.5)	183.9 (180.0–187.7)	5%	0.002
Other	8	5	0.2 (0.1–0.3)	0.1 (0.0–0.2)	-11%	0.18
**Ambulance transport**						
Air	69	88	1.6 (1.2–2.0)	1.8 (1.5–2.2)	4%	0.19
Combined	87	102	2.0 (1.6–2.4)	2.1 (1.7–2.5)	2%	0.46
Ground	4291	5724	99.6 (96.6–102.5)	119.3 (116.3–122.4)	5%	0.002
None	1371	1796	31.8 (30.1–33.5)	37.4 (35.7–39.2)	4%	0.06
Missing	1649	2589	38.3 (36.4–40.1)	54.0 (51.9–56.1)	9%	0.000
**Discharge disposition**						
Inpatient rehabilitation^§^	1101	1371	25.5 (24.0–27.1)	28.6 (27.1–30.1)	3%	0.02
Long-term care facility	1274	1961	29.6 (27.9–31.2)	40.9 (39.1–42.7)	8%	0.001
Home	2779	3600	64.5 (62.1–66.9)	75.1 (72.6–77.5)	4%	0.004
Home with support services	956	1614	22.2 (20.8–23.6)	33.7 (32.0–35.3)	11%	0.005
Died	1192	1596	27.7 (26.1–29.2)	33.3 (31.6–34.9)	5%	0.03
Other^||^	165	157	3.8 (3.2–4.4)	3.3 (2.8–3.8)	-4%	0.46

Abbreviations: TBI, traumatic brain injury; ICISS, ICD-based Injury Severity Score; IQR, interquartile range

* Per 100,000; calculated using population data from Statistics Canada.

† Tested for trend significance using linear regression analysis.

‡ Cut/pierce; drowning/submersion; firearm; machinery; pedal cyclist, pedestrian, or transport (not motor vehicle crash-related); natural/environmental; other specified; unspecified; and adverse effects.

§ Transferred to facility providing inpatient care (e.g. other acute, sub-acute, inpatient rehabilitation).

|| Transferred to other healthcare facility (e.g. palliative care, hospice), signed out against medical advice, unknown disposition.

### Age- and sex-specific trends

Over the five-year study period, the overall rate of TBI hospitalizations increased significantly among both male (p = 0.001) and female (p = 0.02) elderly adults (Table 1). Among both sexes, the oldest age groups (ages 75 to 84 and 85 or older) experienced the greatest average annual increase in the rate of TBI admissions. In contrast, the 65 to 74 age group showed no significant trend among both males and females (p = 0.05 and 0.26, respectively). Altogether, the age of TBI patients increased significantly from 79.2 to 80.2 years over the five-year period (p = <0.001).

### Trends in comorbidity and injury severity

There was a significant trend towards increasing comorbidity level, with the average CCI increasing from 4.6 to 4.8 during the study period (p = 0.002). There was a non-significant trend towards increasing injury severity as measured by the ICISS (p = 0.07). However, there was a significant increase in the TBI rate among the most severe injury categories (ICISS below 75^th^ percentile; p = 0.001; Table 1), with a nonsignificant change in the least severe category (ICISS above 75^th^ percentile; p = 0.25; Table 1).

### Predictors of in-hospital mortality

Over the 5-year study period, the rate of in-hospital mortality increased by an average of 5% per year from 27.7 to 33.3 per 100,000 (p = 0.03; Table 1 and Fig 1). Univariate analysis failed to show a significant change in the odds of mortality over time (OR, 1.00, 95% CI, 0.97–1.02, p = 0.62; Table 2). However, multivariate analysis showed that the odds of mortality actually decreased by 3% each year after accounting for relevant factors such as increasing age and comorbidity (OR, 0.97, 95% CI, 0.95–0.99, p = 0.0072; Table 2).

**Table 2:** Odds ratio (OR) for in-hospital mortality among hospitalizated TBI patients.

	OR (95% CI)	*p*	Adj OR (95% CI)	Adj p
**Age (vs. 65–74)**				
65–74	1.00		1.00	
75–84	1.54(1.42,1.67)	< .0001	1.31(1.19,1.43)	< .0001
85+	2.23(2.06,2.41)	< .0001	1.73(1.57,1.91)	< .0001
**Gender (vs. female)**				
Female				
Male	1.24(1.16,1.31)	< .0001	1.31(1.23,1.40)	< .0001
**Mechanism of injury (vs. other)**				
Other	1.00		1.00	
Fall	1.15(1.01,1.31)	0.034	0.84(0.74,0.96)	0.013
MVC	0.83(0.71,0.98)	0.028	0.67(0.56,0.80)	< .0001
Struck	0.52(0.39,0.69)	< .0001	0.47(0.35,0.63)	< .0001
**CCI (vs. 1–2)**				
1–2	1.00		1.00	
3–4	1.61(1.39,1.88)	< .0001	1.34(1.13,1.58)	0.0006
5+	3.23(2.78,3.74)	< .0001	2.56(2.16,3.04)	< .0001
**ICISS (vs. Above 75th percentile)**				
Below 25th percentile	4.98(4.51,5.50)	< .0001	5.22(4.71,5.78	< .0001
25th to 50th percentile	2.99(2.70,3.30)	< .0001	2.90(2.62,3.22)	< .0001
50th to 75th percentile	1.89(1.69,2.12)	< .0001	1.79(1.60,2.01)	< .0001
Above 75th percentile	1.00		1.00	
**Ambulance transport (vs. none)**				
None	1.00		1.00	
Ground	2.28(2.09,2.48)	< .0001	2.03(1.86,2.22)	< .0001
Combined	2.27(1.79,2.88)	< .0001	2.13(1.66,2.73)	< .0001
Air	3.78(2.97,4.82)	< .0001	3.57(2.77,4.60)	< .0001
**Year**	1.00(0.97,1.02)	0.62	0.97(0.95,0.99)	0.0072

Abbreviations: TBI, traumatic brain injury; ICISS, ICD-based Injury Severity Score.

Model performance was assessed, with area under the receiver operating characteristic curve (AUROC) = 0.7143; Wald χ^2^ = 2229.57, p<0.0001.

Survivor and non-survivor groups differed by age, sex, comorbidity level, length of stay, mechanism of injury, and injury severity (Table 3). In particular, nonsurvivors were more likely to be male, older, have a higher comorbidity level and longer length of stay, sustain a fall-related TBI, and suffer more severe injuries compared to survivors.

**Table 3:** Comparison of characteristics among survivors and non-survivors.

	Survivors, n (%)	Non-survivors, n (%)	p*
**Overall**	36884 (100)	6939 (100)	
**Age (years)**			
65–74	11158 (30)	1332 (19)	< .0001
75–84	15274 (41)	2807 (40)	
85+	10452 (28)	2800 (40)	
**Gender**			
Female	18044 (49)	3042 (44)	< .0001
Male	18840 (51)	3897 (56)	
**Mechanism of injury**			
Fall	30032 (81)	5933 (86)	< .0001
Struck	885 (2)	85 (1)	
MVC	3677 (10)	540 (8)	
Other	2290 (6)	381 (5)	
**CCI**			
0	0	0	
1–2	3047 (8)	253 (4)	< .0001
3–4	17947 (49)	2478 (36)	
5+	15890 (43)	4208 (61)	
**Length of stay (days)**			
1	4275 (12)	1620 (23)	< .0001
2–3	5573 (15)	1079 (16)	
4–6	6203 (17)	1005 (14)	
7–14	8696 (24)	1320 (19)	
15–30	6307 (17)	959 (14)	
30+	5828 (16)	956 (14)	
**ICISS**			
Below 25th percentile	8128 (22)	2810 (40)	< .0001
25th to 50th percentile	10857 (29)	2313 (33)	
50th to 75th percentile	8031 (22)	1092 (16)	
Above 75th percentile	9868 (27)	724 (10)	
**Ambulance transport**			
None	275 (1)	100 (1)	< .0001
Air	426 (1)	93 (1)	
Combined	20332 (55)	4453 (64)	
Ground	6982 (19)	671 (10)	
Missing	8869 (24)	1622 (23)	
**Age,** median (IQR)	79 (12)	83 (12)	< .0001
**CCI,** median (IQR)	4.0 (2.0)	5.0 (2.0)	< .0001
**Length of Stay (days),** median (IQR)	8.0 (17.0)	6.0 (14.0)	< .0001
**ICISS,** median (IQR)	0.8 (0.1)	0.8 (0.1)	< .0001

Abbreviations: TBI, traumatic brain injury; ICISS, ICD-based Injury Severity Score; IQR, interquartile range.

*p-values represent Chi-Square tests comparing survivors/non-survivors for categorical values.

Univariate logistic regression showed that older age, male sex, higher comorbidity level, greater injury severity, and falls were associated with increased odds of in-hospital mortality (Table 3). Multivariate analysis revealed similar findings, with the exception that falls were associated with reduced odds of mortality compared to other mechanisms of injury (OR, 0.84, 95% CI, 0.74–0.96, p = 0.013). Patients 85 years and older had the highest risk of in-hospital mortality with nearly twice the risk as those between 65 and 74 years of age (OR, 1.73, 95% CI, 1.57–1.91, p<0.0001). Males had a 31% higher probability of dying (OR, 1.31, 95% CI, 1.23–1.40, p<0.001) compared to females. Patients with the highest comorbidity level (CCI above 5) had the highest odds of in-hospital death (OR, 2.56, 95% CI, 2.16–3.04, p<0.001). Furthermore, patients with the most severe TBI (ICISS below 25^th^ percentile) had more than five times the risk of mortality (OR, 5.22, 95% CI, 4.71–5.78, p<0.001) compared to those in the least severe category (ICISS above 75^th^ percentile).

### Predictors of Fall-related TBI

**Fig 2:**
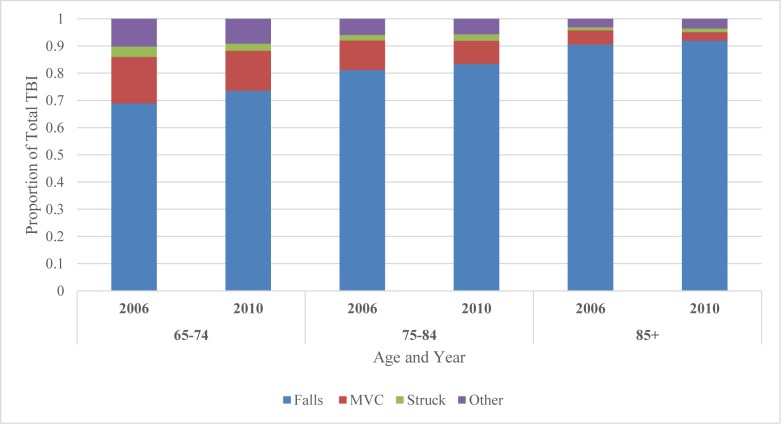
Relative proportions of TBI based on age group, year, and mechanism of injury, HMDB, 2006–2007 to 2010–2011.

Falls were the leading cause of TBI in the elderly, accounting for 82% of TBI hospitalizations and 86% of in-hospital mortality (Table 1 and Fig 2). Over the five-year study period, the number of fall-related TBI admissions increased 44% from 5,975 to 8,595 (Table 1). The rate of falls also increased significantly from 138.6 to 179.2 per 100,000 with an annual average increase of 7% (30% overall increase, p = 0.001; Table 1). Among the falls subpopulation, there were trends towards increasing age and comorbidity level (p = 0.005 and 0.003, respectively). Patients with a fall-related TBI tended to be female, older, and suffer a higher comorbidity compared to patients with a non-fall related TBI (Table 4). On univariate analysis, female sex, older age, and higher comorbidity level were associated with increased odds of sustaining a fall-related TBI (Table 5). Multivariate analysis showed a comparable trend for all the variables. Patients 85 years or older had almost four times the risk of falls compared to those 65 to 74 years old (OR, 3.99, 95% CI 3.68–4.33, p<0.001). Similarly, patients with 5 or more comorbidities had over four times the risk of falls compared to those with only 1–2 comorbidities (OR, 4.30, 95% CI, 3.91–4.74, p<0.001). Males had a 50% decreased risk of falls (OR, 0.51, 95% CI, 0.48–0.54, p<0.0001) compared to that of females. Although injury severity was a significant predictor of falls risk, there was no clear trend associated with increasing falls risk and worsening injury severity (OR, 1.68, 95% CI, 1.56–1.81, p<0.0001). Over the five-year study period, the odds of falls increased by 7% every year (OR, 1.07, 95% CI, 1.04–1.09, p<0.0001) after controlling for relevant variables.

**Table 4:** Comparison of characteristics among fall and non-fall patients.

	Falls, n (%)	Non-Falls, n (%)	p*
**Overall**	35965 (100)	7858 (100)	
**Age**			
65–74	8850 (25)	3640 (46)	< .0001
75–84	15023 (42)	3058 (39)	
85+	12092 (34)	1160 (15)	
**Gender**			
Female	18350 (51)	2736 (35)	< .0001
Male	17615 (49)	5122 (65)	
**Charlson Comorbidity Index**			
0	0 (0)	0 (0)	
1–2	2108 (6)	1192 (15)	< .0001
3–4	16029 (45)	4396 (56)	
5+	17828 (50)	2270 (29)	
**Length of stay (days)**			
1	4788 (13)	1107 (14)	0.0044
2–3	5403 (15)	1249 (16)	
4–6	5940 (17)	1268 (16)	
7–14	8184 (23)	1832 (23)	
15–30	6062 (17)	1204 (15)	
30+	5586 (16)	1198 (15)	
**ICISS**			
Below 25th percentile	8837 (25)	2101 (27)	< .0001
25th to 50th percentile	11328 (31)	1842 (23)	
50th to 75th percentile	7734 (22)	1389 (18)	
Above 75th percentile	8066 (22)	2526 (32)	
**Ambulance transport**			
Air	185 (1)	190 (2)	< .0001
Combined	308 (1)	211 (3)	
Ground	20543 (57)	4242 (54)	
None	6388 (18)	1265 (16)	
Missing	8541 (24)	1950 (25)	
**Died**			
No	30032 (84)	6852 (87)	< .0001
Yes	5933 (16)	1006 (13)	
**Age,** median (IQR)	80.55 (8)	75.88 (7)	< .0001
**CCI,** median (IQR)	4.0 (2.0)	4.0 (2.0)	< .0001
**Length of Stay (days),** median (IQR)	8.0 (17.0)	7.0 (15.0)	0.74
**ICISS,** median (IQR)	0.8 (0.1)	0.8 (0.1)	0.51

Abbreviations: TBI, traumatic brain injury; ICISS, ICD-based Injury Severity Score; IQR, interquartile range.

*p-values represent Chi-Square tests comparing survivors/non-survivors for categorical values.

**Table 5:** Predictors of Fall-induced TBI.

	Odds Ratio (95% CI)	*P*	Adj Odds Ratio (95% CI)	Adj *p*
**Age (vs. 65–74)**				
65–74	1.00		1.00	
75–84	1.51(1.40,1.62)	< .0001	1.98(1.86,2.11)	< .0001
85+	2.42(2.20,2.65)	< .0001	3.99(3.68,4.33)	< .0001
**Gender (vs. female)**				
Female	1.00		1.00	
Male	0.57(0.54,0.61)	< .0001	0.51(0.48,0.54)	< .0001
**Charlson Comorbidity Index (vs. 1–2)**				
1–2	1.00		1.00	
3–4	1.31(1.18,1.44)	< .0001	2.03(1.85,2.22)	< .0001
5+	2.20(1.96,2.46)	< .0001	4.30(3.91,4.74)	< .0001
**ICISS (vs. Above 75th Percentile)**				
Below 25th percentile	1.48(1.36,1.6)	< .0001	1.19(1.10,1.29)	< .0001
25th to 50th percentile	1.84(1.70,2.00)	< .0001	1.68(1.56,1.81)	< .0001
50th to 75th percentile	1.63(1.50,1.78)	< .0001	1.58(1.46,1.72)	< .0001
Above 75th percentile	1.00		1.00	
**Ambulance transport (vs. none)**				
None	1.00		1.00	
Ground	0.87(0.81,0.94)	0.0002	0.96(0.90,1.03)	0.23
Combined	0.34(0.278,0.41)	< .0001	0.29(0.24,0.35)	< .0001
Air	0.22(0.18,0.276)	< .0001	0.19(0.16,0.24)	< .0001
**Year**	1.05(1.03,1.07)	< .0001	1.07(1.04,1.09)	< .0001

Abbreviations: TBI, traumatic brain injury; ICISS, ICD-based Injury Severity Score. Model performance was assessed, with area under the receiver operating characteristic curve (AUROC) = 0.6939; Wald χ^2^ = 2145.3938, p<0.0001.

## Discussion

Elderly populations are at increased risk of TBIs, particularly those caused by falls, and are known to experience worse outcomes and prolonged recovery following these injuries compared to younger adults. Our study examined trends in TBI-related hospitalizations among elderly populations and identified key predictors of TBI-related falls and mortality. We found significant increasing trends in the rates of TBI-related hospitalization and mortality among elderly adults over the five-year study period from 2006/2007 to 2009/2010. The study population also demonstrated significant trends towards increasing age and comorbidity. Falls were the most common and fastest growing mechanism of TBI. Multivariate analysis showed that increasing age, comorbidity, and injury severity were independently predictive of both TBI-related falls and mortality in our study population.

Comparison of TBI rates to the literature is challenging given the paucity of research focusing on elderly populations, and the wide variability in rates by time period and geographical location. Chan et al. studied trends in TBI hospitalizations among elderly adults in Ontario, Canada, over a comparable period from 2003 to 2010. Their study reported overall increased rates of TBI among all elderly adults, ranging from 11% for 65–74 year olds, to 28% for those aged 85 and older. Other studies over the past two decades from Finland, Australia, and the United States have reported similar increases in TBI rates among the elderly ranging from 7% to 34%. [2,5,27,28] Our study described a similar trajectory in TBI rates, finding an average increase of 6% per year (24% overall increase) between 2006 and 2011. These findings highlight the importance of monitoring epidemiological trends in TBI in order to identify at risk populations and develop targeted measures to reduce injury morbidity and mortality.

Our multivariate analysis showed that increased age, comorbidity, and injury severity were independent predictors of mortality, findings which are consistent with many reports in the literature. [29–34] Advanced age, even within the elderly subpopulation, is a known factor associated with mortality following a TBI. McIntyre et al. conducted a meta-analysis of 24 studies published up to July 2011 that reported mortality rates following a TBI. This study included seven studies (n = 15,489) that reported mortality rates by age group, and found that elderly adults aged 75+ were at 1.7 times increased risk of dying compared to those aged 65–74. Another study by Utomo et al. used multivariate analysis to identify predictors of mortality among 428 elderly TBI patients, and found that the 75+ age group had nearly three times the risk of dying compared to those aged 65–74. Utomo et al. also found that severe TBI (Glasgow Coma Scale [GCS] 3 to 8) was associated with a 24-fold increased risk of death compared to mild TBI (GCS 13 to 15). Similarly, McIntyre et al. analyzed 13 studies totaling 35,157 patients, and reported a 12.7 times increased risk of death for severe TBI versus mild TBI.

Comorbidity level has also been shown to be a significant predictor of mortality. Colantonio et al. examined TBI hospitalizations from 1999 to 2002 in Ontario, Canada, and found that having two or more comorbidities more than doubled the risk of mortality compared to those having no comorbidities. [10] Other research has also shown that multiple comorbidities contribute to prolonged recovery and delayed rehabilitation following injury. [4,35] These findings are particularly alarming given the concurrent trends of increasing age and comorbidity identified in this study. As the elderly population continues to expand, inpatient hospitals, rehabilitation centers, and long-term facilities must be prepared to manage growing numbers of increasingly complex elderly patients.

In our study, falls were the most common cause of TBI among elderly adults and experienced the greatest increase over time, a finding that has been well-documented in other studies.[3,5,7,10,11] In our study population, falls accounted for 82% of TBI hospitalizations, a figure that is consistent with the literature, which attribute 61% to 90% of TBIs due to falls.[2,5,8,10,28] Additionally, we reported a 7% average annual increase (30% overall increase) in the rate of fall-related TBIs, which is also consistent with rate increases reported in the literature, which range from 20% to 58%.[2,8,27,36]

There are several possible explanations for the dramatic increase in falls. Firstly, the rise in fall rates could reflect growing public awareness of falls among the burgeoning elderly population, and improved detection rates among physicians as a result of wider access to diagnostic imaging.[36] Secondly, advancements in medical care and overall quality of life in developed countries are allowing elderly adults to live longer with increased comorbidities. The presence of virtually any chronic comorbidity increases the risk of mechanical injury such as falls, particularly among the frail elderly. Our study reported a significant trend towards increasing comorbidity, which is consistent with other studies in the literature.[3,37,38] Furthermore, older age, even in the absence of comorbidities, is associated with an increased risk of falls due to physiologic age-related changes in balance, vision and hearing, physical strength, gait, dexterity, and cognitive skills.[39] These findings are consistent with our study, which found that increased age and comorbidity were significantly predictive of a fall-related TBI on multivariate analysis. Lastly, elderly adults are particularly susceptible to suffering iatrogenic effects of medications and, moreover, are at increased risk of polypharmacy, defined as the simultaneous use of multiple medications such as anticoagulants, psychotropics, and sedatives. Common medication side effects such as dizziness, hypotension, arrhythmias, and decreased level of consciousness can precipitate a mechanical injury in the older adult.[40]

Numerous fall prevention guidelines published by geriatrics organizations and other studies have advocated for multicomponent interventions based on individual fall risk assessments. [39,41,42] Interventions recommended by these guidelines include the following: exercise programs aimed at improving strength and balance, home safety assessments to modify risk hazards, vision assessments to correct poor eyesight, educational programs to encourage safer behavior among patients and families, periodic medication review to eliminate unnecessary medications or mitigate iatrogenic effects, and adequate nutritional supplementation (e.g. vitamin D and calcium) to prevent osteopenia and injury sequelae. However, there is some controversy in the literature regarding the optimal combination of interventions, as well as the cost-effectiveness of single- versus multi-interventional programs.[39,43] In fact, several studies have found that single interventions such as exercise can be equally effective as multifactorial interventions with the benefit of decreased costs and easier implementation.[44–46] Further research is needed to validate the effectiveness of the various aforementioned interventions, and identify the most effective and widely available approach(es) for preventing falls among elderly populations.

The findings of this study must be interpreted in the context of certain limitations. This study was based on administrative data from the HMDB database, which may not capture certain groups at risk for TBI such as prisoners or aboriginal people served by federal agencies. Our data are also subject to potential miscoding, particularly given the number of hospitalizations (14% of admissions) coded as ‘‘other unspecified head injuries” (S09.7 to S09.9), which may include admissions for other TBI or non-TBI diagnoses. In addition, this study is restricted to hospitalized patients, and does not capture milder TBIs identified in outpatient settings such as emergency departments or physician offices

## Conclusion

This study examined nationwide trends in hospitalizations and in-hospital mortality among the elderly across a five-year horizon. A secondary goal of this paper was to identify significant predictors of TBI-related falls and mortality within this vulnerable population. Our study found that the rate of TBI hospitalizations increased significantly over the five-year period, with concurrent trends towards increasing age and comorbidity level. On multivariate analysis, advanced age, comorbidity, and injury severity were independent predictors of both falls and mortality among elderly adults with TBI. Prevention efforts should be targeted to the vulnerable demographics identified in this study, in particular the “older old” (ages 85 and older) and those with multiple medical comorbidities. In addition, inpatient hospital and long-term care facilities should be prepared to manage the burgeoning population of older patients with more complex comorbidities.
